# Accelerometer-Measured Daily Steps, Physical Function, and Subsequent Fall Risk in Older Women: The OPACH Study

**DOI:** 10.1093/geroni/igab046.1725

**Published:** 2021-12-17

**Authors:** Benjamin Schumacher, John Bellettiere, Michael LaMonte, Andrea LaCroix

**Affiliations:** 1 University of California, San Diego, San Diego, California, United States; 2 University of California, San Diego Herbert Wertheim School of Public Health and Human Longevity Science, La Jolla, California, United States; 3 University at Buffalo, Buffalo, New York, United States

## Abstract

We sought to investigate the association between steps per day (steps/d) and incident fall risk while also assessing the role of physical functioning on this association. Steps/d were measured by accelerometer for 7 days in 5,545 women aged 63 to 97 years between 2012 - 2014. Falls were ascertained from daily fall calendars for 13 months. Median steps/d were 3,216. There were 5,473 falls recorded over 61,564 fall calendar months. The adjusted incidence rate ratio comparing women in the highest vs. lowest step quartiles was 0.71 (95% confidence interval, 0.54 - 0.95; P-trend across quartiles of steps/d = 0.01). After further adjustment for physical function using the Short Physical Performance Battery, the rate ratio was 0.86 (0.64-1.16; P-trend = 0.27). Mediation analysis estimated that 66.7% to 70.2% of the association of steps/d and fall risk may be mediated by physical function. In conclusion, higher steps/d were related to lower incident falls primarily through their beneficial association with physical functioning. Interventions that improve physical function, including those that involve stepping, could reduce falls in older adults.

